# Maleic acid and malonic acid reduced the pathogenicity of *Sclerotinia sclerotiorum* by inhibiting mycelial growth, sclerotia formation and virulence factors

**DOI:** 10.1007/s44154-023-00122-0

**Published:** 2023-11-13

**Authors:** Yu-chen Fei, Qin Cheng, Huan Zhang, Chuang Han, Xu Wang, Yan-feng Li, Shi-qian Li, Xiao-hu Zhao

**Affiliations:** 1 State Key Laboratory of Agricultural Microbiology / College of Resources and Environment, Huazhong Agricultural University, Wuhan, 430070 China; 2 Fujian Universities and Colleges Engineering Research Center of Modern Facility Agriculture, Fuqing, 350300 China; 3 Institute of Quality Standard and Monitoring Technology for Agro-Products of Guangdong Academy of Agricultural Sciences, Guangzhou, 510640 China

**Keywords:** *Sclerotinia sclerotiorum*, Maleic acid, Malonic acid, Inhibition

## Abstract

*Sclerotinia sclerotiorum* is a necrotrophic plant pathogenic fungus with broad distribution and host range. Bioactive compounds derived from plant extracts have been proven to be effective in controlling *S. sclerotiorum*. In this study, the mycelial growth of *S. sclerotiorum* was effectively inhibited by maleic acid, malonic acid, and their combination at a concentration of 2 mg/mL, with respective inhibition rates of 32.5%, 9.98%, and 67.6%. The treatment of detached leaves with the two acids resulted in a decrease in lesion diameters. Interestingly, maleic acid and malonic acid decreased the number of sclerotia while simultaneously increasing their weight. The two acids also disrupted the cell structure of sclerotia, leading to sheet-like electron-thin regions. On a molecular level, maleic acid reduced oxalic acid secretion, upregulated the expression of *Ss-Odc2* and downregulated *CWDE10*, *Ss-Bi1* and *Ss-Ggt1*. Differently, malonic acid downregulated *CWDE2* and *Ss-Odc1*. These findings verified that maleic acid and malonic acid could effectively inhibit *S. sclerotiorum*, providing promising evidence for the development of an environmentally friendly biocontrol agent.

**Supplementary Information:**

The online version contains supplementary material available at 10.1007/s44154-023-00122-0.

## Introduction

*Sclerotinia sclerotiorum*, a cosmopolitan necrotrophic pathogen, is a saprophytic and parasitic fungus that infects more than 400 dicotyledons such as sunflowers, soybean, canola and oilseed rape (Chen et al. 2022; Kim et al. 2011; Shahoveisi et al. 2022). Sclerotinia stem rot (SSR) caused by *S. sclerotiorum* occurs in many areas, resulting in a severe yield loss of oilseed rape in China, Canada, the United States and other regions (Bolton et al. 2006; Hu et al. 2019). SSR reduced the annual output of oilseed rape by 10%-30% and even 80% in extreme cases, which seriously endangered agricultural production and caused economic losses (Hu et al. 2017; Qin et al. 2011). Since SSR is a soil-borne disease, the formation of sclerotia in soil plays a significant role in the pathogenic process (Cheng et al. 2019). There are two different approaches for *S. sclerotiorum* to infect the host plant: the main way is hyphae formed directly from germinating sclerotia, and another is through the germinated ascospores (Ding et al. 2021). Pathogenic factors are responsible for the successful infection of *S. sclerotiorum*. Researches have shown that *S. sclerotiorum* releases oxalic acid (OA) to help its colonization of oilseed rape (Ghosh et al. 2016; Fujinami et al. 2022). In the early stage of infection, high concentrations of OA create a reducing environment that can inhibit the oxidative burst of plants and facilitate fungal invasion (Kim et al. 2011). In contrast, low concentrations of OA induce resistance in plants. Therefore, the sclerotial formation and OA secretion of *S. sclerotiorum* are vital for the pathogenic process.

Utilizing chemical pesticides has long been an effective method for preventing and controlling *S. sclerotiorum* (Liu et al. 2018, 2021; Oliveira et al. 2013a, b). However, the current issues of pesticide reduction and fungicide-resistant strains of *S. sclerotiorum* have received considerable attention (Sun et al. 2018; Besil et al. 2018; Zhou et al. 2014a, b). Although numerous pieces of research focus on alternative methods like agricultural practice, biological methods and breeding disease-resistant cultivars (Alvarez et al. 2012; Grandini et al. 2022; Zhang et al. 2020), these methods are not always available and effective. Botanical pesticides are an emerging component of modern pesticide development (Coman et al. 2013; Zhao et al. 2022; Ngegba et al. 2022). Recently, secondary metabolites, such as organic acid, alkaloids and phytosterol, have been used as the main active ingredients of new botanical pesticides, which are biodegradable, economical and environmentally friendly (Luo et al. 2021; Li et al. 2022; Chen et al. 2011a, b). The application of plant extracts as the main active compounds of pesticides to control fungal diseases has a promising prospect.

In our previous studies, we found that dissolved organic matter derived from oilseed rape straw supplemented with selenium (Se) in soil (RSDOM_Se_) inhibited the mycelial growth of *S. sclerotiorum* (Jia et al. 2020, 2019; Cheng et al. 2020). Among the eight metabolites upregulated in RSDOM_Se_, maleic acid and malonic acid inhibited the mycelial growth of *S. sclerotiorum* effectively (Jia et al. 2019). However, there was no report on the effects of the two acids on morphological and physiological characteristics, and relevant pathogenic gene regulations of *S. sclerotiorum* were unknown. To further elucidate the potential inhibitory effects of the two acids on *S. sclerotiorum*, experiments were conducted: (1) to examine the impacts of maleic acid and malonic acid on the antifungal sensitivity, mycelial growth, the pathogenicity of mycelia on detached leaves, sclerotial formation and subcellular structure of sclerotia of *S. sclerotiorum*, and (2) to quantify oxalic acid (OA) secretion in mycelia and assess the expression of relevant pathogenic genes.

## Results

### Effect of maleic acid and malonic acid on the growth of *S. sclerotiorum*

In this study, we clearly clarified the sensitivity of *S. sclerotiorum* to maleic acid and malonic acid (Fig. S1), with the half-maximal effective concentrations (EC_50_) for maleic acid and malonic acid determined to be 2.6 mg/mL and 7.0 mg/mL, respectively. The following studies utilized the effective concentration of 2 mg/mL, which exhibited lower toxicity.

As shown in Table 1, the mycelial growth of *S. sclerotiorum* was significantly inhibited by maleic acid, as well as malonic acid. The inhibition ratios of the three treatments, namely maleic acid (32.5%), malonic acid (9.98%) and maleic acid + malonic acid (67.6%), were determined in comparison to the control. Additionally, the combination of maleic acid and malonic acid effectively inhibited the lesion diameters on detached leaves of oilseed rape. The inhibition ratios were 6.22% for maleic acid, 12.44% for malonic acid, and 20.73% for maleic acid + malonic acid, when compared with the control.

**Table 1:** The lesion diameters of *S. sclerotiorum* determined after 48 h incubation on PDA media with maleic acid and/or malonic acid and the lesion diameters of *S. sclerotiorum* determined after 36 h incubation on detached leaves

Object	Treatments	Lesion length (cm)	Inhibition ratio (%)
Mycelia	Control	8.42 ± 0.09a	0
	Maleic acid	5.68 ± 0.04c	32.5
	Malonic acid	7.58 ± 0.06b	9.98
	Maleic acid + Malonic acid	2.73 ± 0.08d	67.6
Detached leaves	Control	1.93 ± 0.04a	0
	Maleic acid	1.81 ± 0.11a	6.22
	Malonic acid	1.69 ± 0.12ab	12.44
	Maleic acid + Malonic acid	1.53 ± 0.03b	20.73

Data were analyzed by one-way ANOVA and shown as means ± standard error (SE). The concentrations of maleic acid, as well as malonic acid, were 2 mg/mL. Different letters indicated statistically significant differences (*p* < 0.05)

### Inhibitory effect on sclerotial formation

**Fig. 1:**
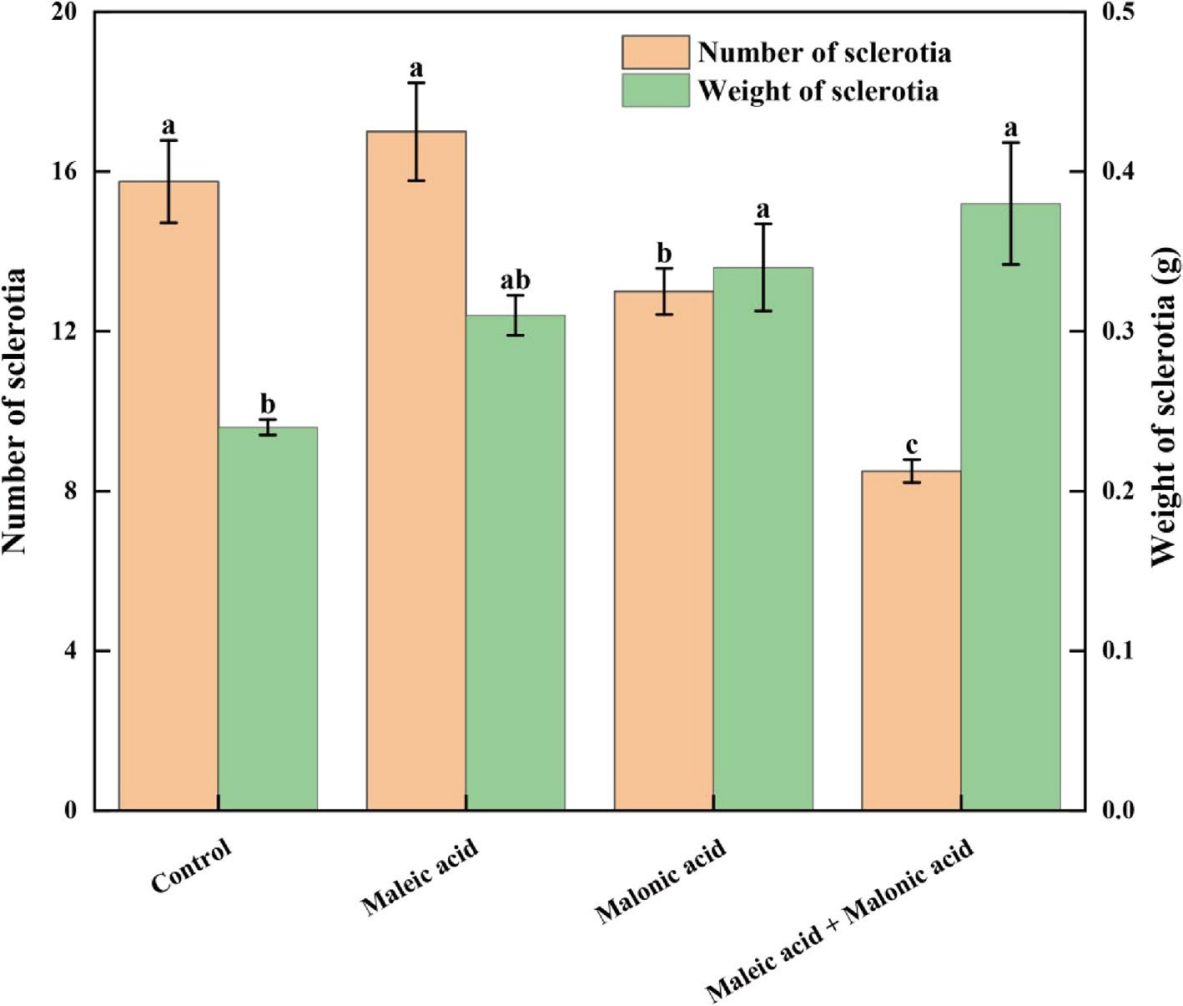
Effects of maleic acid and malonic acid (2 mg/mL) on the number and weight of sclerotia. Data for each column were the per number and weight of sclerotia in one PDA plate. Data were analyzed by one-way ANOVA and shown as mean ± standard error (SE). Different letters indicated statistically significant differences among the different treatments (*p* < 0.05) by Duncan’s tests

The sclerotial formation was examined (Fig. 1). The results indicated sclerotial formation was inhibited by malonic acid, leading to a decrease in the number of sclerotia. However, an increase was observed in their  weight (Fig. 1). Compared with the control, the weight of sclerotia in the treatments of malonic acid and the two-acid combinations increased by 40% and 58%, respectively. The reduction ratios of sclerotial numbers with the two treatments were 17% for malonic acid, and 46% for maleic acid + malonic acid  in comparison to the control. However, the treatment with maleic acid increased both the number and the weight of sclerotia, although these changes were not statistically significant.

### Effect of the two acids on the ultrastructure of sclerotia

**Fig. 2:**
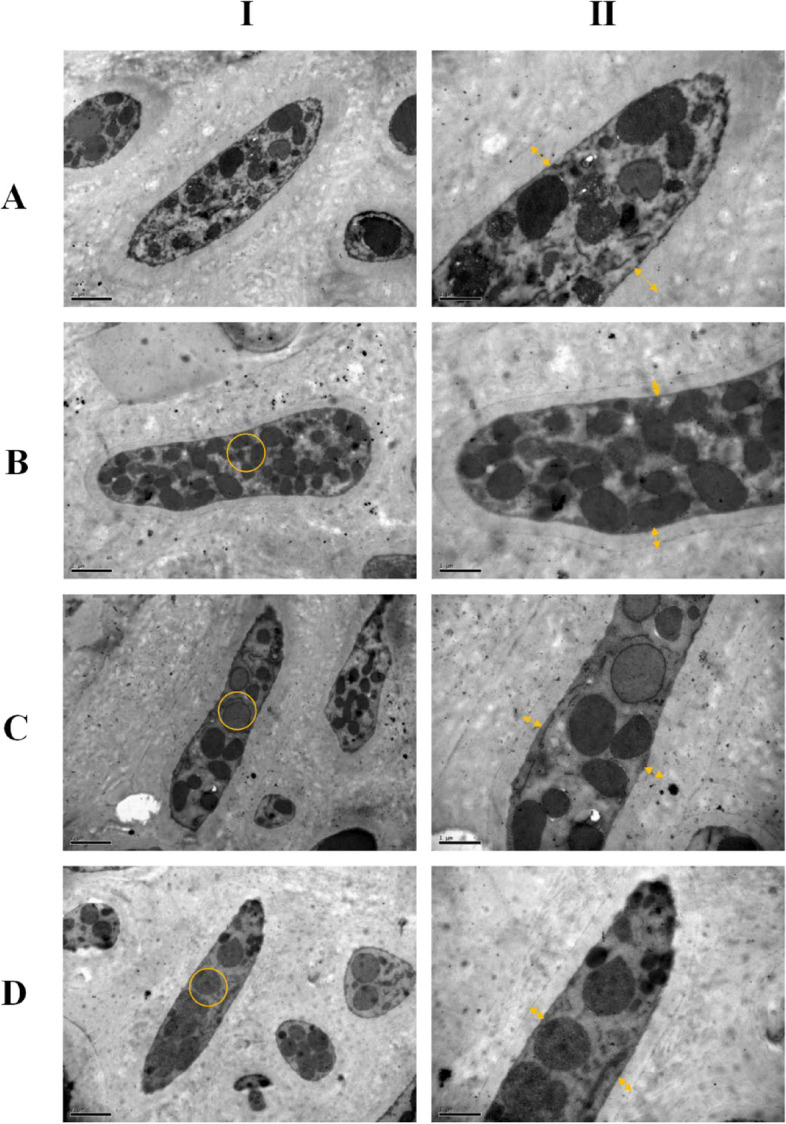
Effects of maleic acid and malonic acid on ultrastructural changes of sclerotia. Representative TEM images of sclerotia sections selected from four specimens in each treatment: **A** The control; **B** 2 mg/mL maleic acid; More particles were formed in sclerotia and different contents reduced. **C** 2 mg/mL malonic acid; Fewer and bigger particles were formed and also the contents degraded. **D** 2 mg/mL maleic acid + 2 mg/mL malonic acid. (I: bar = 2 μm; II: bar = 1 μm). The cell wall became thinner in treatments of the acids, compared with the control. Yellow circles were to mark the changes of contents in sclerotia. The thickness of the cell wall was indicated via yellow arrows

The internal structure sclerotia was observed using TEM. Both acids negatively affected sclerotia compared to the control. In normal sclerotia cells, the cytoplasm exhibits uniformity, the organelles are distinctly visible, and the electron density within the cytoplasm is consistently distributed. (Fig. 2A). After acid treatment, the matrix was sparse and exhibited uneven electron density. The integrity of the cell membrane was compromised, leading to the emergence of multiple patchy regions with reduced electron density within the cell (Fig. 2B, C, and D). In addition, the cell wall became thinner after acid treatment (Fig. 2B, C, and D). Overall, the cellular structure remained largely intact with only a small amount of localized damage observed.

### Analysis of OA secretion and acid production in mycelia

**Fig. 3:**
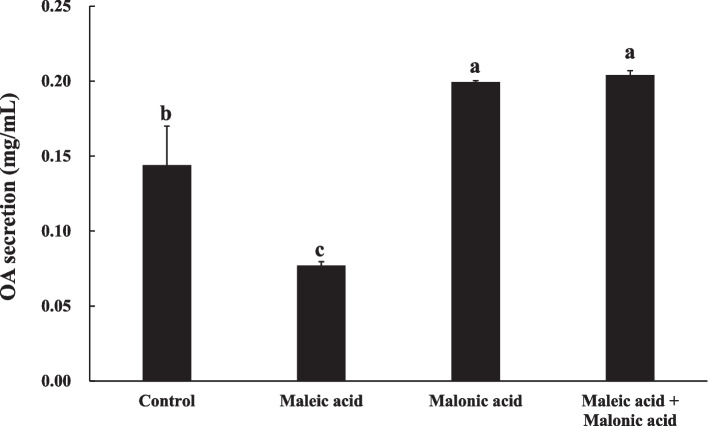
Effect of maleic acid and malonic acid on OA secretion of *S. sclerotiorum*. Data were analyzed by one-way ANOVA and shown as mean value ± standard error (SE). The values with the same letter were not significantly different at *p* < 0.05 according to Duncan’s tests

The OA secretion in mycelia with different treatments was shown in Fig. 3. The corresponding standard curve was shown in Fig. S2, and the R^2^ value of which reached 0.9993. Compared with the control, maleic acid significantly reduced OA secretion, whereas malonic acid treatment and maleic acid + malonic acid treatment significantly increased OA secretion. The decreased ratio of maleic acid on the OA secretion was 45%, and the increased ratios for the treatments of malonic acid and maleic acid + malonic acid were 42% and 46% respectively. pH of maleic acid, malonic acid and their combination in PDB were 2.53, 2.24 and 2.12 respectively. Low pH of the two acids were related to lower pathogenicity of *S. sclerotiorum*.

### qRT-PCR verification of the target gene expression levels

**Fig. 4:**
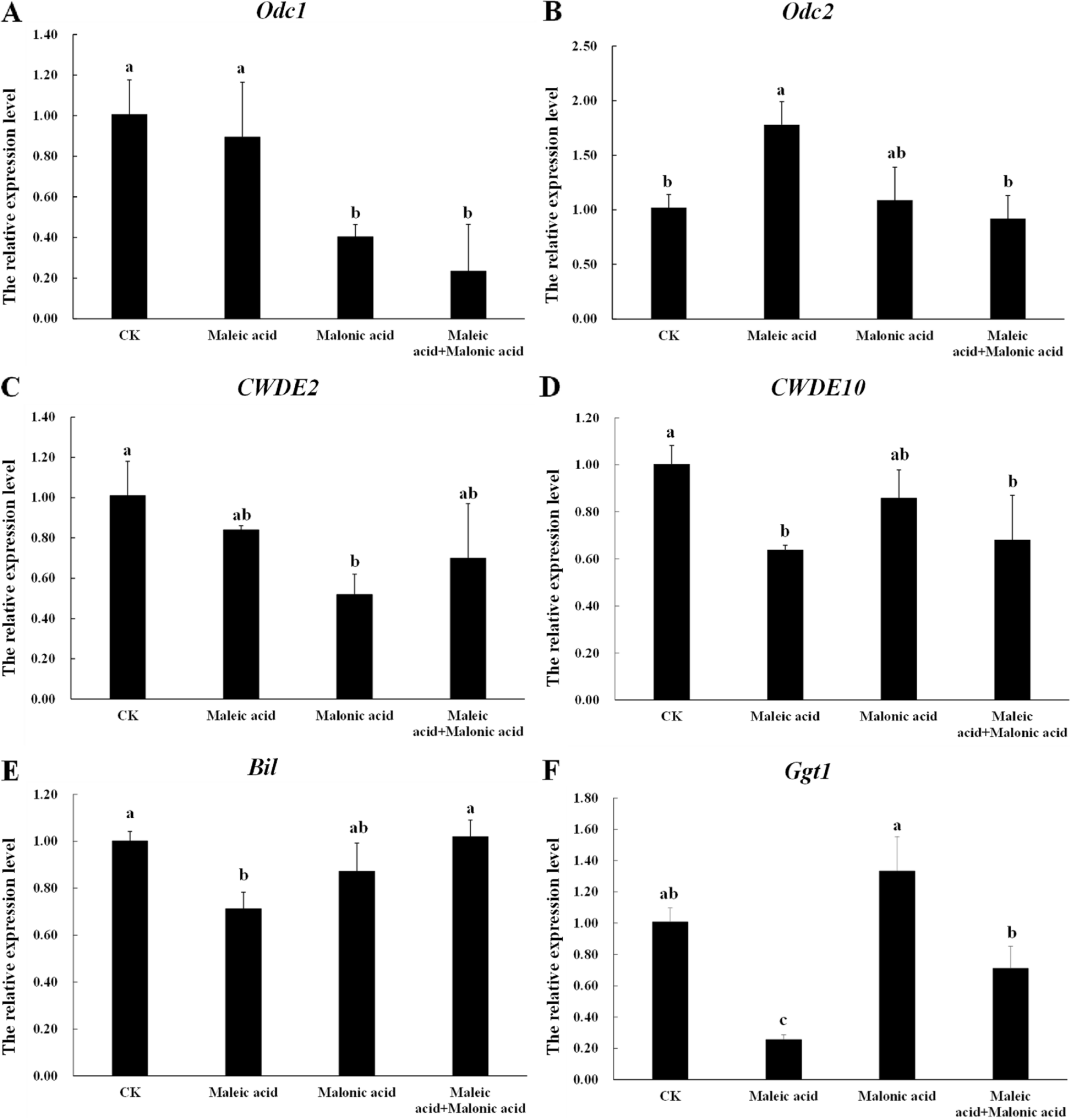
Relative expression levels of six target genes of *S. sclerotiorum*. *S. sclerotiorum* was incubated for 48 h in PDA medium containing different treatments, and mycelia was collected for qRT-PCR analysis. The concentrations of maleic acid, as well as malonic acid, were 2 mg/mL. Data were analyzed by one-way ANOVA and shown as mean value ± standard error (SE). Bars with different letters are significantly different (*p* < 0.05)

Two oxalate decarboxylase (*OxDC*) genes (*Ss-Odc1*, *Ss-Odc2*), two cell wall degradation enzymes (*CWDE2*, *CWDE10*) and two genes related to virulence (*Ss-Bi1*, *Ss-Ggt1*) were evaluated by qRT-PCR. The treatments of malonic acid and the two-acid combination significantly decreased the relative expression level of *Ss-Odc1*, and maleic acid upregulated the expression level of *Ss-Odc2*, as shown in Fig. 4A and B. The treatments of maleic acid and the two-acid combination significantly lowered the expression of *CWDE10* with the corresponding ratios of 36% and 32%, while malonic acid significantly downregulated the expression of *CWDE2* (Fig. 4C and D). As for *Ss-Bi1*, maleic acid decreased the gene expression by 29%, compared with the control. In addition, the expression of *Ss-Ggt1* was declined in the treatments of maleic acid and two-acid combination, with the maleic acid treatment resulting in a 75% decrease (Fig. 4E and F).

## Discussion

**Fig. 5:**
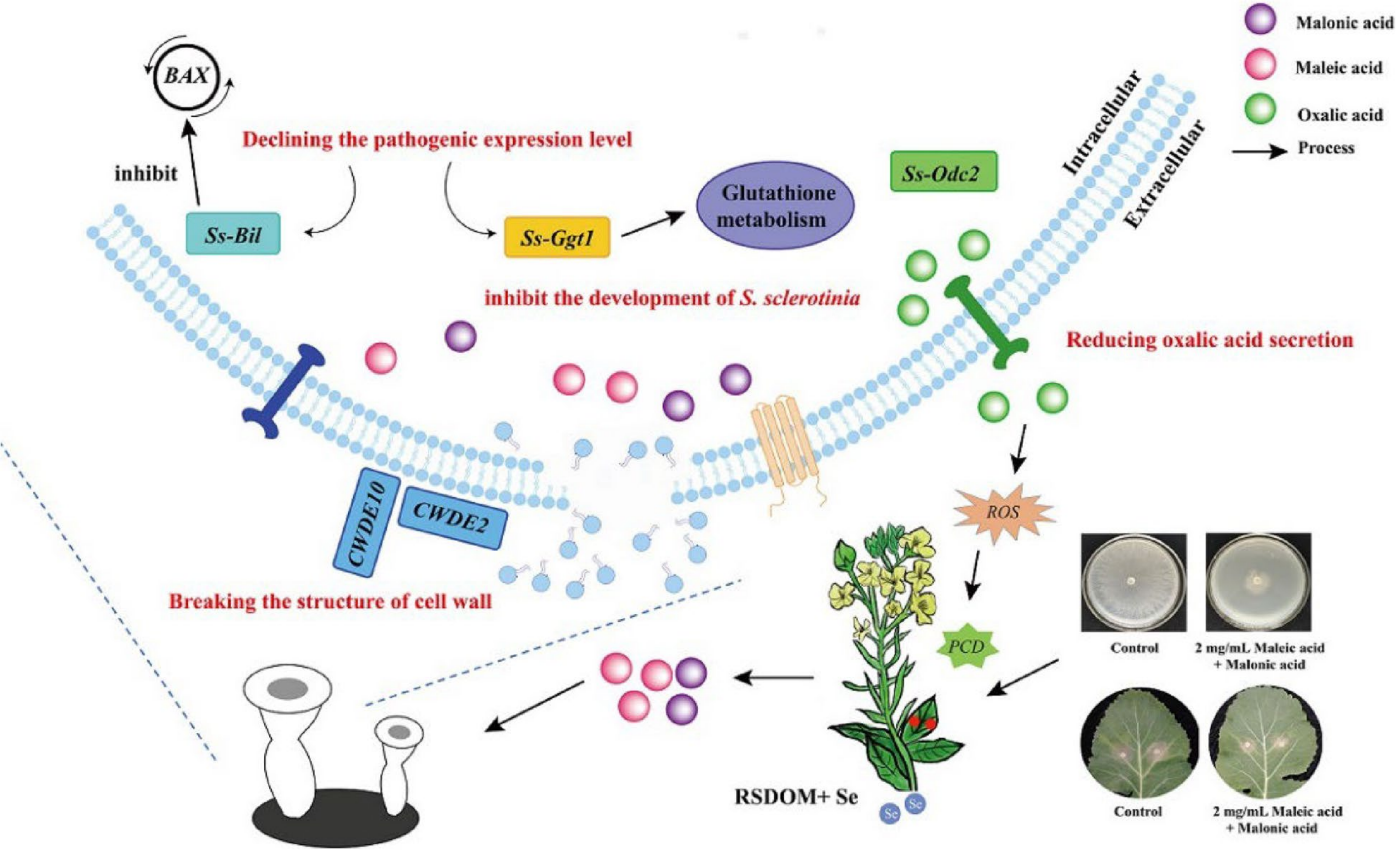
The evidence of inhibition of in vitro growth of *S. sclerotiorum* by maleic acid and malonic acid

Long-term use of traditional pesticides has been found to be detrimental to environment, human health and the progress of ecologically sustainable development (Zhou et al. 2014a, b; Sahni et al. 2016). To reduce the usage of conventional fungicides, alternative methods are worth more attention. In our previous study, it has been proved that RSDOM_Se_ can inhibit the mycelial growth of *S. sclerotiorum*. Maleic acid and malonic acid, which was among the upregulated metabolites of RSDOM_Se_, showed significant inhibitory effect on mycelial growth (Jia et al. 2019). Maleic acid is an important intermediate in chemical industries (Ayoub et al. 2022). It is usually utilized as an acidic catalyst in the food processing industry, due to its non-toxic nature and ediblility (Zhang et al. 2022a, b). Malonic acid is a common component of many products and processes in the pharmaceutical and cosmetic industries (Gu et al. 2022). Studies have shown that malonic acid and maleic anhydride or related compounds have definite antibacterial effects (Chen et al. 2011a, b; Kuwaki et al. 2002). Based on the previous findings, this study provided some evidences that maleic acid and malonic acid inhibit the growth of *S. sclerotiorum* in vitro (Fig. 5).

### Maleic acid and malonic acid reduced the pathogenicity of *S. sclerotiorum*

The activities of fungicides on various plant pathogenic fungi followed the principle of hormesis, described as high-dose inhibition and low-dose stimulation (Zhang et al. 2019). To ensure effective inhibition, the EC_50_ values of the two acids on *S. sclerotiorum* were determined. EC_50_ of maleic acid and malonic acid were 2.6 and 7.0, respectively. Yeon et al. found that maleic acid exhibited antifungal activity against a diverse range of fungi and oomycetes, with the minimum inhibitory concentration ranging from 312.5 to about 2,500 μg/mL (Yeon et al. 2021). In addition, the previous studies showed that malonic acid at a concentration of 2 mg/L had a significant inhibitory effect on *S. sclerotiorum* (Jia et al. 2019). Therefore, the same concentration of 2 mg/mL was selected for this study. Generally, all our designated concentrations stayed within the stimulation phase, and the inhibitory effect of maleic acid was better than that of malonic acid (Fig. S1). The two acids significantly inhibited the mycelial growth of *S. sclerotiorum* and reduced the lesion diameters on the detached leaves (Table 1). The inhibitory effect of the combined application of two acids surpassed that of a single acid treatment. Therefore, it is recommended to utilize a combination of the two acids for the control *S. sclerotiorum*.

### Possible inhibitory evidence regarding the two acids on *S. sclerotiorum*

Further possible inhibitory evidence of maleic acid and malonic acid on *S. sclerotiorum* was also investigated, it might involve the following several processes:

1. The two acids inhibited the sclerotia formation

The sclerotial numbers were significantly reduced at the presence of acombination of two acids, whereas the presence of maleic acid alone resulted in only a slight reduction or no change (Fig. 1). The reduced number of *S. sclerotiorum* suggested that sclerotia were inhibited, corroborating the findings reported by Cheng et al. (2019) and Zhang et al. (2022a, b). Reducing the number of pathogens can effectively mitigate the prevalence of soil-borne diseases (Chen et al. 2011a, b). It is noteworthy that while maleic acid increased both the weight and number of sclerotia (Fig. 1), it significantly inhibited the mycelial growth and the incidence of disease (Table. 1), which may be attributed to the reduction of virulence (Fig. 4). Host-induced gene silencing (HIGS) enhances plant tolerance to pathogens by silencing genes essential for pathogenicity. Zhu et al. found that silencing *CsGPA1* and *CsGPA2* had no impact on the mycelial growth of *S. sclerotiorum*, but it did decrease the quantity of sclerotia and increase the weight of individual sclerotia. Interestingly, only the strain with CsGPA1-silenced exhibited reduced virulence (Zhu et al. 2021). Additionally, a study showed a positive correlation between sclerotinia virulence and colony diameter, but no correlation was found between virulence and the number, size, or weight of sclerotia. (Rather et al. 2022). Consequently, the relationship between the sclerotia formation and virulence of *S. sclerotiorum* needs to be further investigated.

(2) Maleic acid reduced OA production of *S. sclerotiorum*

The synthesis and secretion of OA at high concentrations by *S. sclerotiorum* is a primary determinant for successful plant infection (Hou et al. 2019). In this study, maleic acid significantly curtailed OA secretion, while malonic acid and the combined treatment of two acids enhanced OA secretion (Fig. 3). OA is a key pathogenic factor of *S. sclerotiorum*, which secretes a large amount of OA during early plant infection to suppress the production of plant reactive oxygen species and promote the colonization and expansion of pathogenic bacteria (Cessna et al. 2000). Decreasing OA production in *S. sclerotiorum* could elevate the pH of surrounding environment, thereby diminishing its pathogenicity (Derbyshire et al. 2021). Interestingly, despite the increased OA secretion by *S. sclerotiorum*, malonic acid alone and the combined treatment of two acids exhibited a positive inhibitory effect. One study found that an activating mutation of the *S. sclerotiorum pac1* gene increased oxalic acid production at low pH but decreased virulence (Kim et al., 2007). Therefore, the reduction of virulence of *S. sclerotiorum* induced by maleic acid and malonic acid might be related not only to OA content but also to the pH change caused by it. Another study showed that the growth of *S. sclerotiorum* was affected by pH. Oxalic acid, citric acid, glutaric acid and tartaric acid inhibited sclerotia formation at pH 1.72, 2, 2.43 and 1.96 respectively, and mycelial growth at pH 1.56, 1.88, 2.3 and 1.9 respectively (Atallah et al. 2020). The pH of 2 mg/mL maleic acid, malonic acid and their combination in PDB were 2.53, 2.24 and 2.12 respectively. Therefore, the addition of maleic acid and malonic acid subjected *S. sclerotiorum* to a highly acidic environment, which inhibited its growth*.*

(3) The two acids regulated pathogenic gene expressions of *S. sclerotiorum*

To better understand the potential mechanisms, we evaluated the molecular level associated with OA production, activities of cell wall degradation enzymes (CWDEs) and virulence of *S. sclerotiorum*. *Ss-Odc1* and *Ss-Odc2* are two putative oxalate decarboxylase (*OxDC*) genes. The transcript of *Ss-Odc1* exhibited significant accumulation in different stages of compound appressorium development and plant colonization. In contrast, the *Ss-odc2* transcript was only significantly accumulated only during the middle and late stages of the compound. Evidence indicates that the expressions of *Odc1* and *Odc2* reduced the accumulation of OA, which was not induced by the low pH of the hyphae or exogenous OA (Liang et al. 2015). In this study, maleic acid upregulated the gene expression of *Odc2*, while malonic acid showed no positive effects on the expression of *Odc1*, *Odc2* (Fig. 4A, B), aligning with the determination of OA secretion (Fig. 3). During the fungal infection in plants, an increased level of cell wall degrading enzymes (CWDEs) enhances the fungal pathogens to colonize plants and cause infection (Kubicek et al. 2014, Sun et al. 2023). *S. sclerotiorum* can produce multiple CWDEs that facilitate host penetration, enhance host tissue maceration, and degrade host cell walls (Oliveira et al. 2013a, b). *CWDE2* (cellulase family protein) and *CWDE10* (pectinesterase A) are two kinds of cell wall-degrading enzyme genes (Xu et al. 2015). In this study, maleic acid and malonic acid reduced the virulence of *S. sclerotiorum* by down-regulating *CWDE10* and *CWDE2* respectively (Fig. 4C, D). Interestingly, some studies reported no relations between the gene expression of CWDEs and the pathogenicity of *S. sclerotiorum* (Anees et al. 2010). It may be that increased CWDE transcripts do not necessarily lead to increased virulence in unfavorable environments, such as high pH, where enzyme activity may not be optimal (Favaron et al 2004). *Ss-Ggt1*, a γ-glutamyl transpeptidase, regulates the ROS antioxidant system (Li et al. 2012). As for *Ss-Bi1*, it encodes a putative *Bax*-inhibitor protein that is vital in the hyphal stress response and full virulence of *S. sclerotiorum*, influencing the pathogenicity in an oxalic acid-independent manner (Yu et al. 2015). The declining gene expression might indicate gene silencing so that *Bax* expression is inhibited and PCD (Programmed Cell Death) could not be activated to enhance plant resistance to pathogens (Shlezinger et al. 2011). However, results of this study showed that only maleic acid facilitate plant resistance against *S. sclerotiorum* through down-regulating *Ggt1* and *Bi1* (Fig. 4E, F).

(4) Role of Maleic Acid in the TCA Cycle Enhances Plant Resistance

**Fig. 6:**
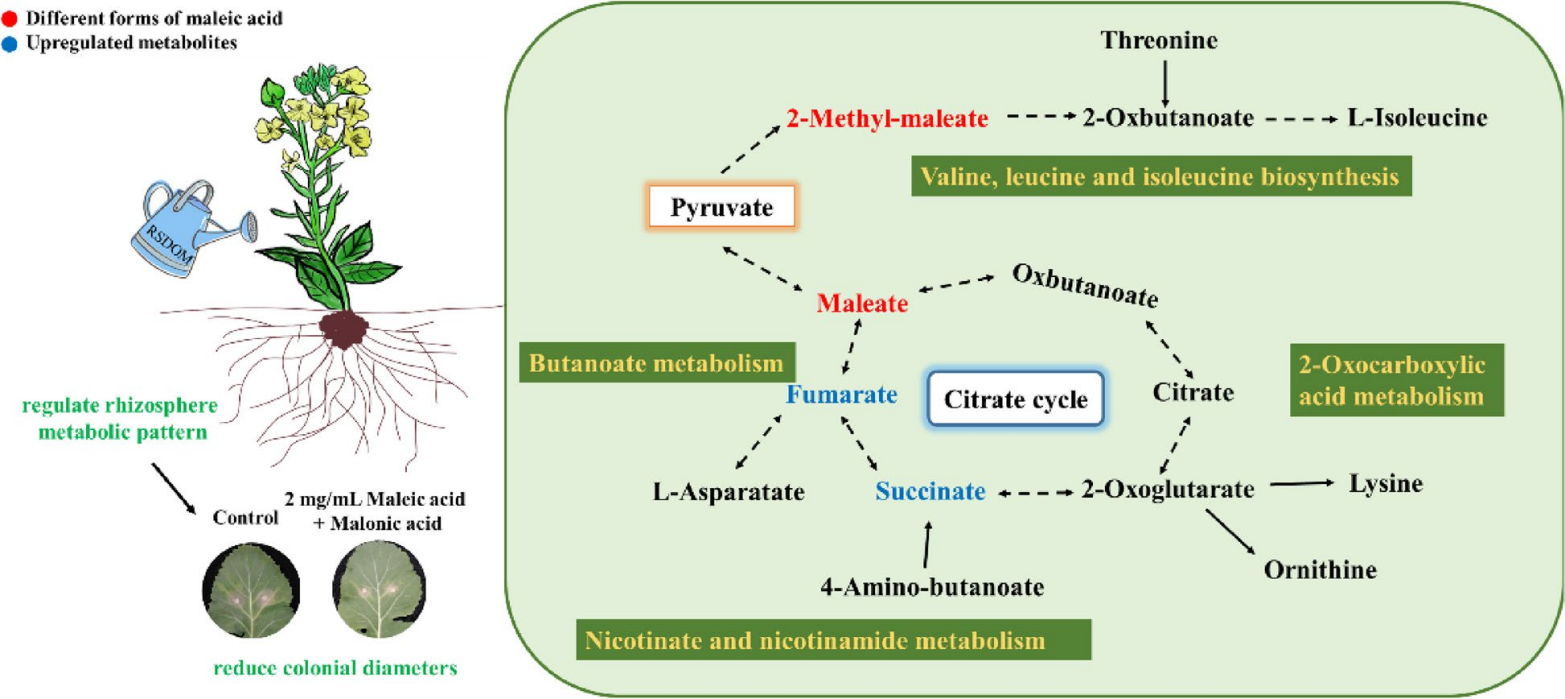
Role of Maleic Acid in the TCA Cycle Enhances Plant Resistance. As revealed by the up-regulated KEGG (Kyoto Encyclopedia of Genes and Genomes) pathway, several metabolic pathways contribute to enhancing plant resistance, with maleic acid participating in some of them such as the TCA cycle

In our previous study, we found the application of RSDOM_Se_ exhibited a significant antifungal effect on *S. sclerotiorum*. According to the analysis of differential metabolites and up-regulated KEGG (Kyoto Encyclopedia of Genes and Genomes) metabolic pathways, the inhibitory effect of RSDOMSe might be associated with the upregulation of not only maleic acid and malonic acid but also metabolic pathways related to maleic acid (Jia et al. 2019). Succinic acid and fumaric acid, two main components of the tricarboxylic acid (TCA) cycle, were identified as two key metabolites that were up-regulated with RSDOMSe treatment (Fig. 6). Some studies have shown that succinic acid had the potential to participate in the host's immune regulation as a signal molecule (Jiang et al. 2023; Wei et al. 2023). Meanwhile, the TCA cycle not only contributes to the maintenance of energy metabolism homeostasis but also promotes the synthesis of non-essential amino acids such as aspartic acid, which can help plants absorb nutrients and maintain metabolic stability (Yang et al. 2021).

## Conclusions

The combination of maleic acid and malonic acid, derived from oilseed rape straw, could effectively control *S. sclerotiorum*. This control is achieved by inhibiting mycelial growth, damaging the subcellular structure of sclerotial, reducing oxalic acid secretion and regulating the expression of pathogenic genes. Malonic acid was effective in inhibiting the mycelial growth and sclerotia formation of *S. sclerotiorum*. Maleic acid, on the other hand, reduced the pathogenicity of *S. sclerotiorum* by decreasing OA secretion and reducing the expression of virulence-related genes such as *Ss-Bi1* and *Ss-Ggt1*. In addition, the detached leaf experiments showed that the combination of the two acids could effectively reduce the infection of *S. sclerotiorum* in oilseed rape. This study suggested that maleic acid and malonic acid had potential as safe ecological inhibitors for *S. sclerotiorum*, which provided a theoretical reference for the subsequent development of green and environmentally friendly pesticides.

## Material and methods

### Pathogen and chemicals

*S. sclerotiorum* (JZJL-13) used in this study was obtained from the Key Laboratory of Crop Disease Monitoring and Safety Control, Huazhong Agricultural University. Fungal strains were cultured on potato–dextrose–agar (PDA) medium (200 g potato, 20 g dextrose, and 15 g agar in 1 L water), and the corresponding liquid medium was potato-dextrose-broth (PDB) medium. Sclerotia were activated at first, and mycelial plugs cut with the same radius were placed into a new PDA and incubated at 23 °C for 48 h to obtain new mycelia of *S. sclerotiorum*. Maleic acid (ID: 392248) and malonic acid (ID:844) used in this study were purchased from Aladdin Reagent limited-liability company in Shanghai.

### Antifungal activity assay

To estimate the activity of *S. sclerotiorum* responding to the two acids*,* the half-maximal effective concentrations (EC_50_) were determined according to Jia et al. (2019). Different gradient concentrations of maleic acid (2, 4, 6, 8, 10 mg/mL) and malonic acid (0.8, 1, 1.6, 2.4, 3.2 mg/mL) were set to measure the mycelial growth of *S. sclerotiorum*. The prepared mycelial plugs (6 mm in diameter) of 2-day-old colonies in PDA media were transferred to PDA media with thegradient concentrations of maleic acid and malonic acid. Culturing *S. sclerotiorum* on PDA with no acid addition was the control treatment. The colony diameters of mycelial agar in the petri dish were determined after incubation in darkness at 23 °C for 48 h. According to Cheng et al. (2019), the inhibition ratio was defined as follows: “d_control_” was the mycelial colony diameter of *S. sclerotiorum* in the PDA medium, and “d_treated_” was the colony diameter of *S. sclerotiorum* in the PDA medium with maleic acid or malonic acid. Each treatment was repeated four times.

$$\mathrm{Inhibition\,ratio}\left(\mathrm{\%}\right)=\frac{{{d}_{control}-d}_{treated}}{{d}_{control}}\times 100\%$$

The “logit” method was utilized to proceed with nonlinear data fitting. The values in the X-axis refer to the gradient concentrations of the acid, and the values in the Y-axis refer inhibition ratios of the acid (Sebaugh 2011). Based on the results of EC_50_ and low phytotoxicity, an equal concentration of 2 mg/mL was selected for the following study. The fresh mycelial agar was placed on the center of the PDA medium with four treatments: the control, 2 mg/mL of maleic acid, 2 mg/mL of malonic acid, 2 mg/mL of maleic acid + 2 mg/mL of malonic acid (the same as below). Each treatment was preformed with four replicates.

### Estimation of pathogenicity on detached leaves of oilseed rape

The oilseed rape selected in this experiment was *Brassica napus L.* cultivar Zhongshuang No.9 from the Oil Crops Research Institute, Chinese Academy of Agricultural Sciences. Detached leaves of oilseed rape were picked from the eco-agriculture base (30°28′26’’N, 114°2′15’’E), Huazhong Agricultural University, Wuhan, China. Mycelial plugs (6 mm in diameter) with different treatments were inoculated onto the detached oilseed leaves with wounds pretreated with a sterile knife, and the diameters of wounds on the leaves were the same size as the prepared mycelial plugs. The colony diameters of the detached leaves were measured by cross method 36 h later to examine the pathogenicity. Each treatment was repeated four times.

### Sclerotial formation determination

To estimate the effect of maleic acid and malonic acid on sclerotial formation, the numbers and weight of *S. sclerotiorum* in treatments of the two acids were determined. Similarly, mycelial plugs were transferred to fresh PDA media with different treatments. Each petri dish was incubated at 23 °C in darkness for 15 d. Then, the number of sclerotia on each PDA plate was recorded, and the sclerotia were collected and weighed. Each treatment was repeated four times.

### Transmission electron microscopy (TEM) analysis

To study the subcellular effect that maleic acid and malonic acid exerted on *S. sclerotiorum*, TEM observation was considered a priority to observe the ultrastructure of sclerotia, and the operational process was based on Cheng et al. (2019). After collecting sclerotia from the PDA medium with different treatments, sclerotia were fixed in a solution of 2.5% glutaraldehyde in 100 mM phosphate buffer (pH = 7.2) at 4 °C for 4 h. After that, phosphate buffer was used to rinse samples for 4 h. Next, two-hour required for the rinsed samples immersed in 1% osmium tetroxide with the same buffer at 4 °C. Then, the samples were dehydrated in graded acetone series for 4 h, completely immersing them in a mixed solution with graded acetone and resin for 4 d. Ultimately, a Leica Ultracut UCT ultramicrotome with a diamond knife was utilized to obtain ultra-thin Sects. (50 nm) of the samples. The samples were finally observed by an electron microscope (TEM, H-7650, Hitachi, Japan).

### Oxalic acid secretion and acid production determination

The OA secretion of *S. sclerotiorum* in the PDB media was determined according to Jia et al. (2019). The 2-day-old mycelial agars were transferred to PDB media with different treatments and were cultured in the dark at 23℃ for 72 h. Each PDB medium had 5 mycelial agars. Afterwards, the PDB solution was centrifuged (10,000 × g, 15 min) to obtain the supernatant. Subsequently, the determination of OA content followed the colorimetric method. 0.4 mL supernatant was moved to a colorimetric tube with 0.1 mL 0.5 mg/mL Fe^3+^ standard solutions (FeCl_3_), 1 mL KCl–HCl solution (3.7 g/L KCl and 5.4 g/L HCl, pH 2.0) and 0.06 mL 0.5% sulfosalicylic acid (w/v). After 20 min, the absorbance at 510 nm was read from a UV-5200 ultraviolet spectrophotometer. The acid of the liquid was determined by the Seven2Go pH meter S2-Std-Kit (Cheng et al. 2019). The pH in the PDB medium was measured to investigate the change in acid production in mycelium due to treatments. Each treatment was repeated four times.

### RNA isolation and quantitative real-time PCR (qRT-PCR) analysis

The determination of the relevant gene expression levels was based on Xu et al. (2020). This experiment included two main steps: acquisition of mycelial samples and specific determination of the gene expression process. To obtain mycelium samples, mycelial plugs were inoculated onto sterilized cellophane disks on PDA plates for 48 h at 23 °C. After that, the mycelia on the cellophane were collected and ground with high-throughput tissue grinding machines (Jingxin Corporation, Shanghai). The determination process was mainly divided into three parts, including extraction of RNA, reverse transcription of RNA, and quantitative PCR detection. Mycelial RNA was extracted according to NI-*Sclerotinia sclerotiorum* RNA Reagent (Newbio Industry, Tianjin, China), and RNA samples were reversely transcribed by EasyScript One-Step gDNA Removal and cDNA Synthesis SuperMix (TransGen Biotech, Beijing) to obtain cDNA. Quantitative PCR detection was performed using the ABI Q6 Flex system (Applied Biosystems, USA). Target primer sequences were listed in Table S1 (Supplementary). The reference gene, *β-tublin,* was used to normalize the transcript levels of target genes. Each qRT-PCR was repeated three times and each biological replicate had two technical replicates. The 2^− ΔΔCT^ method was applied for determining the expression of target genes.

### Statistical analysis

All data analyses were performed with SPSS software version 22.0. Data preprocessing included the test of Normality test and homogeneity of variance. After that, one-way analysis of variance (ANOVA) was adopted for a series of experiments including antifungal sensitivity assay, estimation of pathogenicity on detached leaves of oilseed rape, sclerotial formation determination, OA secretion determination, RNA isolation, and quantitative real-time PCR (qRT-PCR) analysis. Duncan’s test was to compare the means of the treatments. When *p* < 0.05, the result was considered significant.

### Supplementary Information

**Additional file 1.**

## Data Availability

The datasets analyzed during the current study are available from the corresponding author upon reasonable request.

## Abbreviations

RSDOM_Se_: Dissolved organic matters derived from rape straw supplemented with selenium in soil
*S. sclerotiorum*: *Sclerotinia sclerotiorum*
PCR: Polymerase Chain Reaction
TEM: Transmission Electron Microscopy
RT-qPCR: Reverse Transcription Quantitative Real-time Polymerase Chain Reaction
OA: Oxalic acid
SSR: Sclerotinia stem rot
PCD: Programmed cell death
