# Supplementary material for: Maleic acid and malonic acid reduced the pathogenicity of Sclerotinia sclerotiorum by inhibiting mycelial growth, sclerotia formation and virulence factors
Source: Stress Biol. 2023 Nov 13;3(1):45. doi: 10.1007/s44154-023-00122-0 (PMC10643788; doi:10.1007/s44154-023-00122-0)
Supplement: Supplementary file 1 — Additional file 1. [file 44154_2023_122_MOESM1_ESM.docx]

**Table S1 qPCR primer sequence of target gene**

| Gene name | Forward primer(5'-…-3') | Reverse primer(5'-…-3') | Amplification efficiency |
| --- | --- | --- | --- |
| *Ssodc1* | GATGAGGGCCAACTTACGGT | CGGGAGTGTTAGGCTTCAGG | 95.5% |
| *Ssodc2* | ATTTCTTGCCAACGCCCAAC | TGGGCCACGTAAGTTTGGAA | 96.9% |
| *CWDE2* | ATGCTCCTTTCACCACAACC | ACACCCCCATTCGCATAATA | 95.2% |
| *CWDE10* | CCAATGGAGATCAGCAAGGT | TTGTACATTCGCCAAACCAA | 103.1% |
| *SsBi1* | TCATTCCAGCAAACCTACAACC | GAACATAGCACCACCCACGAG | 102.3% |
| *SsGgt1* | AGATCGCCCAACTTCACTCG | TTCCCTCTGTCAAAGTCGCC | 97% |
| *β-tublin* | TTGGATTTGCTCCTTTGACCAG | AGCGGCCATCATGTTCTTAGG | 100.5% |

**Fig. S1 Baseline sensitivity of mycelial growth.**

Note: Different gradient concentrations of maleic acid (2, 4, 6, 8, 10 mg/mL) and malonic acid (0.8, 1, 1.6, 2.4, 3.2 mg/mL) were set up to measure the mycelial growth of sclerotia under these concentrations. The virulence regression equation of maleic acid was y = 7.1897x - 2.9667, y = 0, x = 2.57, EC_50_ = 2.57 mg/mL, *p* < 0.001 (a); For malonic acid was y = 5.0832x-4.3213, y = 0, x = 7.08, EC_50_ = 7.08 mg/mL, *p* < 0.001 (b).

**Fig. S2 The standard curve used for oxalic acid determination**
